# Clinical Outcomes for Patients With Metastatic Breast Cancer Treated With Immunotherapy Agents in Phase I Clinical Trials

**DOI:** 10.3389/fonc.2021.640690

**Published:** 2021-03-17

**Authors:** Anna R. Schreiber, Jodi A. Kagihara, Jennifer A. Weiss, Andrew Nicklawsky, Dexiang Gao, Virginia F. Borges, Peter Kabos, Jennifer R. Diamond

**Affiliations:** ^1^ Department of Medicine, University of Colorado Anschutz, Aurora, CO, United States; ^2^ Department of Medicine, University of Colorado Cancer Center, Aurora, CO, United States; ^3^ University of Colorado School of Medicine, Aurora, CO, United States

**Keywords:** immunotherapy, metastatic breast cancer, PD-L1 inhibitors, phase I clinical trials, PD-1 inhibitors

## Abstract

**Background:**

Immuno-oncology (IO) agents have demonstrated efficacy across many tumor types and have led to change in standard of care. In breast cancer, atezolizumab and pembrolizumab were recently FDA-approved in combination with chemotherapy specifically for patients with PD-L1-positive metastatic triple-negative breast cancer (TNBC). However, the single agent PD-1/PD-L1 inhibitors demonstrate only modest single agent efficacy in breast cancer. The purpose of this study was to investigate the efficacy of novel IO agents in patients with metastatic breast cancer (MBC), beyond TNBC, treated in phase I clinical trials at the University of Colorado.

**Methods:**

We performed a retrospective analysis using a database of patients with MBC who received treatment with IO agents in phase I/Ib clinical trials at the University of Colorado Hospital from January 1, 2012 to July 1, 2018. Patient demographics, treatments and clinical outcomes were obtained.

**Results:**

We identified 43 patients treated with an IO agent either as a single agent or in combination. The average age was 53 years; 55.8% had hormone receptor-positive/HER2-negative breast cancer, 39.5% TNBC and 4.7% HER2-positive. Patients received an average of 2 prior lines of chemotherapy (range 0-7) in the metastatic setting. Most patients (72.1%) received IO alone and 27.9% received IO plus chemotherapy. Median progression-free survival (PFS) was 2.3 months and median overall survival (OS) was 12.1 months. Patients remaining on study ≥ 6 months (20.9%) were more likely to be treated with chemotherapy plus IO compared to patients with a PFS < 6 months (77.8% v. 14.7%). No differences in number of metastatic sites, prior lines of chemotherapy, breast cancer subtype, absolute lymphocyte count, or LDH were identified between patients with a PFS ≥ 6 months vs. < 6 months.

**Conclusions:**

Our phase I experience demonstrates benefit from IO therapy that was not limited to patients with TNBC and confirms improved efficacy from IO agents in combination with chemotherapy. A subset of patients with MBC treated in phase I clinical trials with an IO agent derived prolonged clinical benefit. Predictors of response to immunotherapy in breast cancer remain uncharacterized and further research is needed to identify these factors.

## Introduction

Breast cancer is the most common cancer in women and patients with metastatic breast cancer (MBC) have a 5-year overall survival of only 27% (1). While prognosis depends on biologic subtype, there remains a critical unmet need for novel therapeutic options to improve survival for patients with MBC.

The development of immuno-oncology (IO) therapeutics has changed the way we treat many cancers, most dramatically with inhibitors of programmed cell death-1 (PD-1), its ligand (PD-L1) and cytotoxic T-lymphocyte-associated antigen (CTLA-4) (2–4). The first approval in metastatic breast cancer came in 2019 with the approval of atezolizumab in combination with nab-paclitaxel for patients with PD-L1-positive (tumor-infiltrating immune cells ≥ 1%) metastatic triple-negative breast cancer (TNBC) (5). This was followed in 2020 by the approval of pembrolizumab in combination with chemotherapy including paclitaxel, nab-paclitaxel or gemcitabine plus carboplatin in patients with PD-L1-positive (combined positive score ≥10) metastatic TNBC (5, 6).

TNBC and human epidermal growth factor receptor 2 (HER2)-positive breast cancers are perceived as being more immunogenic compared to luminal breast cancers based on a higher mutational burden, higher tumor-infiltrating lymphocyte (TIL) rates and higher PD-L1 expression (7–10). Higher TIL expression is associated with increased pathologic complete response (pCR) rates in patients treated with neoadjuvant chemotherapy and improved prognosis in HER2-positive and TNBC (9, 11). PD-L1 is expressed in 20-50% of breast cancers and varies depending on the specific antibody clone and evaluation on tumor cells or immune cells in the tumor microenvironment (10). Expression is higher in TNBC and HER2-positive breast cancer compared to hormone receptor (HR)-positive/HER2-negative tumors (10, 12). In patients with TNBC and HER2-positive breast cancer treated with neoadjuvant chemotherapy, PD-L1 expression correlates with a higher pCR rates and improved clinical outcomes (13–15).

Despite the increased immunogenicity of TNBC, response rates to IO monotherapy with pembrolizumab range from 23% for PD-L1-positive patients treated in the first-line setting to approximately 5% for patients previously treated with chemotherapy regardless of PD-L1 status (16, 17). While there is a subset of patients with TNBC who are exceptional responders to immunotherapy and experience long-term disease control, the efficacy of IO monotherapy generally is no better than palliative chemotherapy and combinations of IO plus chemotherapy are more active (5, 6, 18).

The activity of IO agents in luminal breast cancers is more limited with response rates ranging from 11% to 30% for pembrolizumab in patients with advanced PD-L1 positive, HR-positive HER2-negative breast cancer and 3% with avelumab in a similar patient population (19, 20). In the neoadjuvant setting, the addition of pembrolizumab to an anthracycline and taxane-containing chemotherapy backbone resulted in an increased pCR rate in patients with HR-positive/HER2-negative breast cancer in the I-SPY2 clinical trial (21). There are numerous ongoing clinical trials evaluating IO agents in combination with chemotherapy, radiotherapy, other immune checkpoint inhibitors or cancer vaccines (22).

Over the last decade, there has been a rapid increase in the development of diverse IO agents targeting numerous pathways extending beyond CTLA-4 and PD-1/PD-L1. The recent approval of atezolizumab and pembrolizumab in combination with chemotherapy for patients with PD-L1-positive TNBC allows for an IO option for a subset of patients with metastatic breast cancer. Outside of this limited indication, the opportunity for many patients to receive treatment with an IO agent has been in the setting of a clinical trial. Given the great enthusiasm for IO agents in general for the treatment of cancer and the promise of durable responses for some patients, we observed high enrollment of patients with metastatic breast cancer in phase I clinical trials evaluating IO agents at our site.

The purpose of this study was to evaluate clinical outcomes for patients with metastatic breast cancer who were treated in phase I clinical trials containing at least one IO agent at the University of Colorado Cancer Center. We included patients with all breast cancer subtypes who were treated with many different types of IO agents ranging from PD-1/PD-L1-inhibitors to cancer vaccines. While each phase I trial enrolled a small number of patients with metastatic breast cancer, we sought to combine these patients into one dataset to explore outcomes for IO agents in a phase I breast cancer population.

## Materials and Methods

We performed a retrospective analysis using a database of patients from the electronic medical record system (EMRS) with MBC who received treatment with IO agents in phase I/Ib clinical trials at the University of Colorado Hospital from January 1, 2012 – July 1, 2018. All data was stored in a secure online database and the study was performed in accordance with local IRB guidelines. Phase I trials included all protocols that studied single agent or multi-agent investigational drugs that had phase I or phase Ib in the title. For patients in phase Ib/II trials, only patients enrolled in the phase Ib portion of the study were included for analysis. Patients were included in the study if they received an agent considered to directly target or modulate immune cells or immune cell signaling (an IO agent).

Patient characteristics including age, sex, presence of metastatic disease at diagnosis, number of sites of metastases, lines of prior systemic therapy, HR and HER2 receptor status, Eastern Cooperative Oncology Group Performance Status (ECOG PS), radiation within 30 days of IO and mean lab values were collected *via* chart review using the EMRS. HR and HER2 receptor status was based on local pathology report also found in the EMRS. Other data collected included: time of treatment discontinuation, disease progression and death. We did not collect PD-L1 status as this was not uniformly performed for all patients with MBC during the time period of this study at our institution.

Investigational treatments were administered at the University of Colorado Hospital as part of a clinical trial that received institutional review board (IRB) approval. All patients provided written informed consent prior to enrollment in these phase I clinical trials.

### Endpoints and Statistical Methodology

Cohort characteristics were summarized using counts with percentages for categorical variables and with the mean with quartiles for continuous variables. The association between cohort characteristics and progression-free survival (PFS) was evaluated with the Wilcoxon rank-sum test for continuous variables and the Fisher Exact test for categorical variables due to low cell counts. The Wilcoxon rank-sum test was chosen to account for the non-normal distribution of the continuous variables.

PFS was defined as the time from study enrollment to the date of discontinuation for progressive disease, initiation of a new anti-cancer therapy, or death. Overall survival (OS) was defined as the time from study enrollment to the date of death. Patients lost to follow-up were censored at the last follow-up date. For patients who remained on study, the date of analysis (May 1^st^, 2019) was used to censor the patient outcomes. The median number of months for OS and PFS were calculated using the Kaplan-Meier method with *p*-values determined by log-rank test. *p*-values were reported based on a null hypothesis of no difference against a two-sided alternative. Analyses were performed using SAS 9.4 (SAS Institute; Cary, NC).

## Results

### Baseline Patient Characteristics

A total of 43 patients with MBC were treated with a wide range of IO agents in phase I/Ib clinical trials at the University of Colorado Hospital during the period of our study. The average age was 53 years (range 33-71) and all patients were female (**Table 1**). ECOG PS was 0 in 53.5% of patients and 1 in 46.5% of patients. Most patients had 3 or more sites of metastasis (51.6%). On average, patients received two prior lines of chemotherapy (range 0-7) in the metastatic setting. Most patients had HR-positive/HER2-negative breast cancer (55.8%), followed by TNBC (39.5%) and HER2-positive disease (4.7%). In the phase I/Ib clinical trials, 72.1% of patients received single or combination immunotherapy and 27.9% received an IO agent plus chemotherapy.

**Table 1:** Baseline Patient Characteristics.

	Total Patients	PFS <6 months	PFS ≥ 6 months	p-value
Total Number Patients (N)	43	34	9	–
Age (years)				
Mean	52.58	52.71	52.11	0.9167^±^
Range	(33-71)	(33-71)	(39-62)	
Sex				
Male	0 (0%)	0 (0%)	0 (0%)	–
Female	43 (100%)	34 (100%)	9 (100%)	
Metastatic disease at diagnosis	3 (7.14%)	3 (9.09%)	0 (0%)	1.0000^*^
Number of Metastatic Locations				
1	9 (20.93%)	6 (17.65%)	3 (33.33%)	0.4284*
2	12 (27.91%)	9 (26.47%)	3 (33.33%)	
3+	22 (51.16%)	19 (55.88%)	3 (33.33%)	
Lines of chemotherapy in metastatic setting				
Mean	2.14	2.09	2.33	0.8179^±^
Range	(0-7)	(0-5)	(0-7)	
Receptor status				1.0000^*^
HR+/HER2-	24 (55.81%)	19 (55.88%)	5 (55.56%)	
HER2+	2 (4.65%)	2 (5.88%)	0 (0%)	
TNBC	17 (39.53%)	13 (38.24%)	4 (44.44%)	
Treatment				
PD-L1/PD-1	12 (27.91%)	11 (32.35%)	1 (11.11%)	0.0015^*^
IO + Chemotherapy	12 (27.91%)	5 (14.71%)	7 (77.78%)	
Other IO, No Chemo	19 (44.19%)	18 (52.94%)	1 (11.11%)	
ECOG PS				
0	23 (53.49%)	18 (52.94%)	5 (55.56%)	–
1	20 (46.51%)	16 (47.06%)	4 (44.44%)	
Radiation within 30 days of IO	3 (6.98%)	1 (2.94%)	2 (22.22%)	0.1060^*^
Lymphocyte count (k/uL)				
Mean (SD)	1.23 (0.68)	1.13 (0.47)	1.59 (1.14)	0.2948^±^
Alkaline Phosphatase (U/L)				
Mean (SD)	99.72 (56.82)	105.5 (62.42)	77.89 (14.18)	0.1389^±^
LDH (U/L)				
Mean (SD)	449.71 (787.83)	500.22 (863.14)	217.4 (99.02)	0.6313^±^

*Fisher Exact Test.

^±^Wilcoxon rank-sun Test.

ECOG PS, Eastern Cooperative Oncology Group Performance Status; SD, Standard Deviation.

Patients with HR-positive/HER2-negative breast cancer all received hormonal therapy prior to enrollment in the phase I/Ib clinical trials (**Supplemental Table 1a**). The most common prior therapy administered in the HR-positive/HER2-negative group, included capecitabine (45.8%) and everolimus (45.8%). Around one-fifth (20.8%) of patients with HR-positive/HER2-negative cancers received a cyclin-dependent kinase (CDK) 4/6 inhibitor prior to enrollment in phase I/Ib clinical trials. A little less than half (44.4%) of HR-positive/HER2-negative patients received a CDK 4/6 inhibitor and one patient (5.6%) received alpelisib following progression on phase I/Ib clinical trials (**Supplemental Table 1b**). In TNBC, the most common prior therapy administered in any setting was carboplatin with gemcitabine (64.7%) and doxorubicin, cyclophosphamide, paclitaxel/docetaxel (47.1%) (**Supplemental Table 1c**). Around one-quarter (23.5%) of patients with TNBC received sacituzumab govitecan prior to enrollment in phase I/Ib trials. The most common therapy received post-progression in TNBC was eribulin (28.6%) (**Supplemental Table 1d**). One patient (7.1%) with TNBC received sacituzumab govitecan following progression. Prior therapies received in HER2-positive cancers are listed in **Supplemental Table 1e**.

### Phase I Clinical Trials Including IO Agents

**Figure 1:**
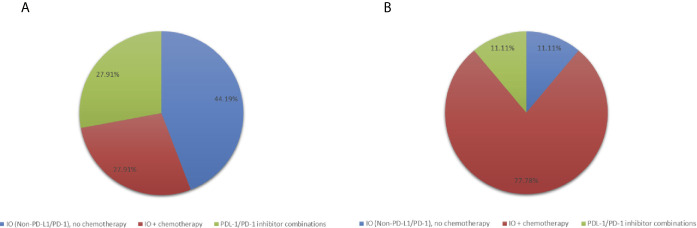
Pie-charts showing **(A)** Treatment distribution in all patients (N=43) **(B)** Treatment distribution in patients with PFS ≥ 6 months (N=9).

In the phase I studies included in this analysis, patients were treated with PD-L1/PD-1 inhibitors without chemotherapy (N=12, 27.9%), IO agents other than PD-L1/PD-1 inhibitors without chemotherapy (N=19, 44.2%) or any IO agent plus chemotherapy (N= 12, 27.9%) (**Figure 1**). Patients treated with PD-L1/PD-1 inhibitors without chemotherapy also received other agents targeting vascular endothelial growth factor (VEGF), indoleamine 2,3-dioxygenase 1 (IDO), OX40, CD38, and T-cell immunoreceptor with Ig and ITIM domains (TIGIT). Trials containing IO agents other than PD-L1/PD-1 inhibitors without chemotherapy included agents targeting IL-10 inhibitor, toll-like receptor 9 (TLR9) agonist and cancer vaccines. Trials with an IO agent in combination with chemotherapy included agents targeting PD-L1, cancer vaccines, nab-paclitaxel, cyclophosphamide and FOLFOX chemotherapy.

### Clinical Outcomes

**Figure 2:**
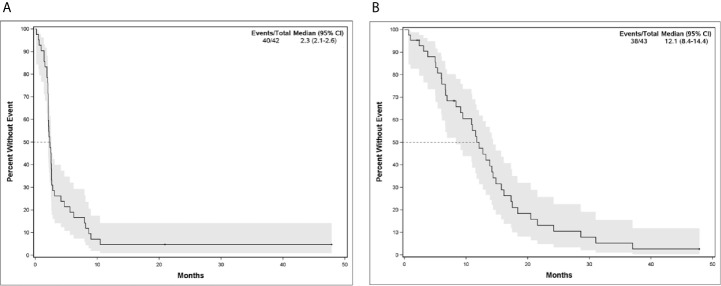
**(A)** Progression-Free Survival and **(B)** Overall Survival for Patients with Metastatic Breast Cancer in Phase I/Ib Clinical Trials Treated with IO Agents.

**Figure 3:**
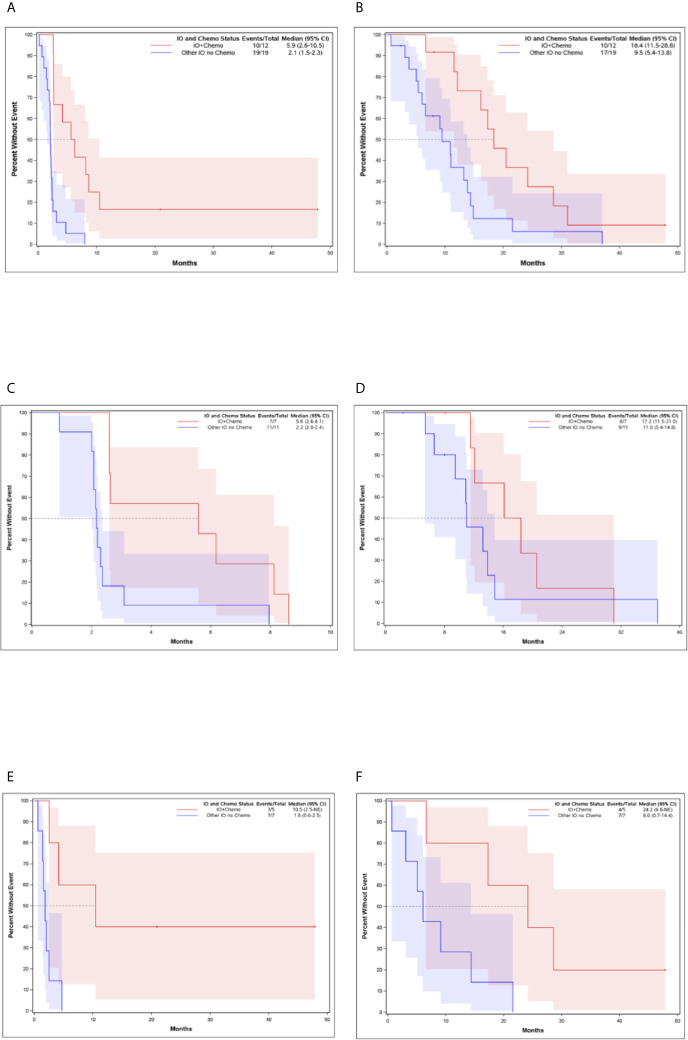
Kaplan-Meier Curves for Patients Who Received IO and Chemotherapy Compared to Patients Who Received IO Without Chemotherapy **(A)** Progression-Free Survival for all patients **(B)** Overall Survival for all patients **(C)** Progression-Free Survival for HR-positive, HER-2 negative breast cancer **(D)** Overall Survival for HR-positive, HER-2 negative breast cancer **(E)** Progression-Free Survival for TNBC **(F)** Overall Survival for TNBC.

The median PFS and OS for all patients with MBC enrolled in phase I clinical trials including any IO agent was 2.3 months (95% CI, 2.07-2.60) and 12.1 months (95% CI, 8.35-14.27), respectively (**Figure 2**). Patients who received an IO agent plus chemotherapy had an improved PFS (5.9 months [95% CI, 2.60-10.45] vs. 2.1 months [95% CI, 1.55-2.30], p<0.001) and OS (18.4 months [95% CI, 11.54-28.60] vs. 9.5 months [95% CI, 5.39-13.84] p=0.015) compared to those who received an IO agent without chemotherapy (**Table 2**, **Figure 3**). In subgroup analysis for patients with HR-positive/HER2-negative breast cancer, median PFS was prolonged in patients treated with IO plus chemotherapy compared to IO alone (5.6 months [95% CI, 2.6-8.1] vs. 2.2 months [95% CI, 2.0-2.4], p = 0.0096) (**Figure 3**). There was also a trend towards improved OS in these patients (17.2 months [95% CI, 11.5-31.0] vs. 11.0 months [95% CI, 5.4-14.8], p= 0.276). Similar findings were observed in patients with TNBC with improved median PFS (10.5 months [95% CI, 2.5- NE (Not Estimable)] vs. 1.8 months [95% CI, 0.6-2.5], p=0.008) and OS (24.2 months [95% CI, 6.6-NE] vs. 6.0 months [95% CI, 0.7-14.4], p=0.0193) in patients treated with IO plus chemotherapy versus IO alone (**Figure 3**).

**Table 2:** Progression-Free Survival (PFS) and Overall Survival (OS) for Patients Who Received IO Plus Chemotherapy Compared to Patients Who Received IO Only.

	IO + Chemotherapy	IO Only	p-value^2^
Median PFS (months)^1^	5.88 (95% CI, 2.60-10.45)	2.07 (95% CI, 1.55-2.30)	<0.001
Median OS (months)	18.38 (95% CI, 11.54-28.60)	9.47 (95% CI, 5.39-13.84)	0.015

CI, confidence interval

^1^One observation dropped due to unknown reason going off-study in calculation of PFS.

^2^p-values generated using log-rank test.

We identified 9 patients (20.9%) with PFS ≥ 6 months which we considered to be consistent with clinical benefit (**Table 1**). Of these, 5 had HR-positive/HER2-negative breast cancer and 4 patients had TNBC. Patients with PFS ≥ 6 months, were treated with IO plus chemotherapy (N=7, 77.8%) and IO alone (N=2, 22.2%) (**Table 1**, **Figure 1**). Patients with PFS ≥ 6 months were more likely to receive an IO agent plus chemotherapy compared to those with PFS < 6 months (77.8% vs. 14.7%). No significant differences in prior lines of therapy, lymphocyte count, alkaline phosphatase, or lactate dehydrogenase (LDH) were identified between patients with PFS < 6 months and ≥ 6 months.

Five patients (11.6%) had PFS ≥ 9 months (range 9 months to >36 months) which we considered to be consistent with durable response. All but one of these patients were treated with IO plus chemotherapy and four of the five patients had TNBC. The best response observed in our study was in a 59-year-old woman with TNBC metastatic to her chest wall, lymph nodes and lungs who was treated with anti-PD-L1 and chemotherapy in the second-line setting. Chemotherapy was discontinued after 4 cycles due to neuropathy and she continued on single agent anti-PD-L1 for another 11 cycles before developing immune-mediated pneumonitis requiring discontinuation of immunotherapy. She had a complete clinical response to therapy and remains with no evidence of disease 3.5 years later.

## Discussion

Our study looked at clinical outcomes in patients with previously-treated metastatic breast cancer treated in phase I clinical trials that included an IO agent. We included patients with all breast cancer subtypes treated with many different IO agents targeting PD-1/PD-L1, but also other immune checkpoints and cancer vaccines. Patients were previously treated with an average of 2 prior lines of chemotherapy in the metastatic setting, approximately 20% of patients with HR-positive/HER2-negative disease previously received CDK 4/6 inhibitors and 23.5% of patients with TNBC received prior sacituzumab govitecan. Our study demonstrates that regardless of breast cancer subtype or specific IO target, patients with metastatic breast cancer (TNBC or endocrine-resistant HR-positive/HER2-negative) treated with combinations of IO plus chemotherapy had prolonged PFS and OS compared to patients treated with IO agents alone. Our study found limited efficacy for IO agents administered without chemotherapy, including novel immune checkpoint inhibitor combinations, in patients with previously-treated metastatic breast cancer.

In our study, the median PFS for all patients with previously-treated metastatic breast cancer who received an IO agent in a phase I clinical trial was a modest 2.3 months. This is consistent with other reports of outcomes for similar patients treated in phase I trials (23). However, a unique finding of our study looking specifically at patients receiving IO agents was the observation of durable responses (PFS ≥ 9 months) in 11.6% of patients including one patient who experienced a durable remission lasting many years after stopping therapy for toxicity. Additionally, 20.9% of patients had PFS ≥ 6 months consistent with clinical benefit. Durable responses to immunotherapy observed in our study are consistent with what has been observed in other larger trials of IO agents in breast cancer and other solid tumors where durable remissions can occur even in patients with widespread metastatic disease (24–26).

The majority of patients in our study who experienced durable long-term responses (PFS > 9 months) were patients with metastatic TNBC treated with IO plus chemotherapy consistent with the now proven benefit of FDA-approved regimens in PD-L1-positive metastatic TNBC. Notably, the efficacy of the combination of atezolizumab and nab-paclitaxel in patients with PD-L1-positive TNBC was confirmed in the phase III first-line Impassion130 trial resulting in FDA-approval following the observation of preliminary efficacy in a Phase Ib trial including previously-treated patients (5, 26). Despite our patients being somewhat heavily pretreated, a subset of patients with TNBC still derived long-term benefit from IO + chemotherapy when treated in a phase I clinical trial setting.

There were patients in our study with metastatic HR-positive/HER2-negative breast cancer, resistant to endocrine therapy, that derived benefit from IO plus chemotherapy including one patient with a durable response (PFS > 9 months) and 5 patients with PFS > 6 months. The efficacy of single agent PD-1/PD-L1-inhibitors in HR-positive HER2-negative breast cancer is modest with response rates lower than in TNBC (16, 17, 19, 20, 27, 28). Our results support the many ongoing clinical trials of IO agents in combination with chemotherapy in patients with endocrine-resistant HR-positive/HER2-negative breast cancer (22).

Clinical benefit in our study was greater in patients treated with IO plus chemotherapy and this finding was observed in patients with both endocrine-resistant HR-positive/HER2- breast cancer and TNBC which is also consistent with other studies in breast cancer demonstrating modest response rates with IO agents alone (16, 17, 19, 20, 26, 29, 30). Patients in our study with HR-positive/HER2-negative breast cancers, had a median PFS of 5.6 vs. 2.2 months (p=0.0096) in those treated with IO plus chemotherapy compared to IO monotherapy. OS was also improved however this result was not statistically significant.

There are few studies which have examined IO agents in combination with chemotherapy in a metastatic HR-positive/HER2-negative population. Interestingly, a recently released study examining survival of HR-positive/HER2-negative MBC patients treated with eribulin with or without pembrolizumab did not find improvement in OS or PFS in the IO plus chemotherapy group, which differs from our findings (31). In the I-SPY2 trial, the combination of pembrolizumab with chemotherapy led to a more than doubling of the pCR rate in patients with early stage HR-positive/HER2-negative cancers who had a MammaPrint that was not in the low risk range (21). The limited efficacy of IO monotherapy in HR-positive/HER2-negative breast cancer has been hypothesized to be potentially related to lower PD-L1 expression, tumor-infiltrating lymphocytes (TILs) and tumor mutation burden (TMB) in this disease subset (9, 31–33). Current data suggest that the addition of chemotherapy to IO agents may have multiple favorable effects including stimulation of the immune system by release of tumor neoantigens and recruitment of antigen-presenting cells (22, 34). Moreover, IO plus chemotherapy combination may delay the development of resistance to treatment (35).

Our study relays the importance of phase I clinical trials, often thought as a last resort for patients with advanced malignancy. Enrollment in phase I clinical trials remains a viable option for select patients with previously-treated metastatic breast cancer (23). Of the patients examined, 11.6% had durable response and one patient with metastatic TNBC remains disease free after 3.5 years. It is estimated that only 3-5% of United States adult cancer patients are enrolled in clinical trials (36). However, when comparing breast cancer (BC) patients to the general population, BC patients appear to obtain clinical benefit from phase I therapies with similar toxicity (36). As a result of phase I trials, atezolizumab with nab-paclitaxel is FDA approved for metastatic TNBC as is tucatinib in the treatment of HER2-positive MBC (5, 26, 37, 38). Phase I trials are important for discovering promising therapies and should continue to be utilized.

While our study showed benefit of IO plus chemotherapy in a metastatic TNBC and HR-positive population, there were several limitations. Limitations to our study included the retrospective nature of the analysis and our inability to include PD-L1-expression as a variable. We included patients treated with a diverse range of IO agents making our population heterogeneous and patients were all treated at a single academic center. There were overall very modest patient numbers and very few patients with HER2-positive breast cancer were treated in these studies. Another limitation to our study was that only a fifth of patients received prior therapy with CDK 4/6 inhibitors. As CDK 4/6 inhibitors are standard first line therapies, our study may not be able to be extrapolated to patients who did receive this therapy prior to IO.

The success of the combination of IO plus chemotherapy in TNBC highlights the potential for activity of new therapies in early phase clinical trials in carefully selected patients. Our study demonstrates that the benefit derived from novel IO agents is not limited to a TNBC population. Despite these benefits, larger, multi-center trials are needed in order to better understand the use of IO agents in all breast cancer subtypes.

## Data Availability Statement

The raw data supporting the conclusions of this article will be made available by the authors, without undue reservation.

## Ethics Statement

The studies involving human participants were reviewed and approved by Colorado Multiple Institutional Review Board. The patients/participants provided their written informed consent to participate in this study.

## Author Contributions

AS, JD: manuscript writing and submission. AS, JD, JK, JW: material preparation and data collection. AN, DG: statistical analysis and figure development. JD, JK, VB, PK: study review and monitoring. All authors contributed to the article and approved the submitted version.

## Funding

This work was supported by the Women’s Cancer Developmental Therapeutics Program at the University of Colorado Cancer Center, Internal Medicine Resident Research Program, University of Colorado School of Medicine, NIH/NCI through 5K23CA172691 (PI Diamond), P30CA046934 (UCCC CCSG), and under Ruth L. Kirschstein National Research Service Award T32CA236734-01 (Kagihara).

## Conflict of Interest

The authors declare that the research was conducted in the absence of any commercial or financial relationships that could be construed as a potential conflict of interest.
