# Supplementary material for: Clinical Outcomes for Patients With Metastatic Breast Cancer Treated With Immunotherapy Agents in Phase I Clinical Trials
Source: Front Oncol. 2021 Mar 17;11:640690. doi: 10.3389/fonc.2021.640690 (PMC8010246; doi:10.3389/fonc.2021.640690)
Supplement: Supplementary file 1 [file Table_1.docx]

S1a: All prior targeted and chemotherapy agents in HR+/HER2- breast cancers in a curative and metastatic setting.

| **HR+/HER2-** | Patients N=24* |
| --- | --- |
| Capecitabine | 11 (45.8) |
| Everolimus | 11 (45.8) |
| Cyclophosphamide with doxorubicin | 10 (41.7) |
| Cyclophosphamide, doxorubicin, paclitaxel/docetaxel | 6 (25.0) |
| Palbociclib | 5 (20.8) |
| Paclitaxel | 4 (16.7) |
| Gemcitabine with carboplatin | 4 (16.7) |
| Sapanisertib | 3 (12.5) |
| Paclitaxel with bevacizumab | 3 (12.5) |
| Cyclophosphamide with docetaxel | 3 (12.5) |
| Eribulin | 3 (12.5) |
| Pegylated liposomal doxorubicin | 2 (8.3) |
| Ixabepilone with capecitabine | 2 (8.3) |
| Nab-paclitaxel | 2 (8.3) |
| Capecitabine with carboplatin | 1 (4.2) |
| Capecitabine with lapatinib | 1 (4.2) |
| Carboplatin, doxorubicin | 1 (4.2) |
| Carboplatin with paclitaxel | 1 (4.2) |
| Carboplatin, docetaxel, veliparib | 1 (4.2) |
| Trastuzumab | 1 (4.2) |
| Etoposide | 1 (4.2) |
| Gemcitabine | 1 (4.2) |
| Gemcitabine with vinorelbine | 1 (4.2) |
| Ixabepilone, gemcitabine, everolimus | 1 (4.2) |
| Nab-paclitaxel with bevacizumab | 1 (4.2) |
| Nab-paclitaxel with everolimus | 1 (4.2) |
| Sacituzumab govitecan | 1 (4.2) |
| Sapanisertib with alisertib | 1 (4.2) |
| Seviteronel | 1 (4.2) |
| Vantictumab with paclitaxel | 1 (4.2) |
| Vinorelbine | 1 (4.2) |

*All patients received hormonal therapy

S1b: Post-progression therapies for patients with metastatic HR+/HER2- breast cancer. Hormone therapy not included.

| **HR+/HER2-** | Patients N=18* |
| --- | --- |
| Palbociclib | 7 (38.9) |
| Sapanisertib | 5 (27.8) |
| Navelbine | 5 (27.8) |
| Capecitabine | 3 (22.2) |
| Everolimus | 3 (22.2) |
| Carboplatin with gemcitabine | 3 (22.2) |
| Eribulin | 3 (22.2) |
| Cyclophosphamide with doxorubicin | 2 (11.1) |
| Paclitaxel | 2 (11.1) |
| Gemcitabine | 2 (11.1) |
| Pegylated liposomal doxorubicin | 2 (11.1) |
| Nab-paclitaxel | 2 (11.1) |
| Abemaciclib | 2 (11.1) |
| Nab-paclitaxel plus gemcitabine | 1(5.6) |
| Olaparib | 1(5.6) |
| Genentech GO39733 vaccine | 1(5.6) |
| Everolimus plus navelbine | 1(5.6) |
| Oratecan plus PGP inhibitor | 1(5.6) |
| Pembrolizumab | 1(5.6) |
| Alpelisib | 1(5.6) |

* Number reflects patients with data on post-progression therapies and excludes patients lost to follow-up or who died prior to initiation of post-progression therapy.

S1c: All prior targeted and chemotherapy agents in TNBC in a curative and metastatic setting.

| **TNBC** | Patients N=17 |
| --- | --- |
| Carboplatin with gemcitabine | 11 (64.7) |
| Cyclophosphamide, doxorubicin, paclitaxel/docetaxel | 8 (47.1) |
| Eribulin | 5 (29.4) |
| Capecitabine | 4 (23.5) |
| Cyclophosphamide with doxorubicin | 4 (23.5) |
| Sacituzumab govitecan | 4 (23.5) |
| Capecitabine with docetaxel | 3 (17.6) |
| Carboplatin with docetaxel | 2 (11.8) |
| Cyclophosphamide with docetaxel | 2 (11.8) |
| ENMD-2076 | 2 (11.8) |
| Ixabepilone | 2 (11.8) |
| Nab-paclitaxel | 2 (11.8) |
| Atezolizumab plus nab-paclitaxel | 1 (5.9) |
| Carboplatin | 1 (5.9) |
| Carboplatin, docetaxel, trastuzumab | 1 (5.9) |
| Cisplatin with rucapirib | 1 (5.9) |
| Cyclophosphamide with epirubicin | 1 (5.9) |
| Cyclophosphamide with epirubicin/fluorouracil | 1 (5.9) |
| Eribulin, trastuzumab, pertuzumab | 1 (5.9) |
| Ixabepilone with capecitabine | 1 (5.9) |
| Trametinib with uprosertib | 1 (5.9) |
| Navicixizumab | 1 (5.9) |
| Navelbine | 1 (5.9) |
| ONT10 vaccine | 1 (5.9) |
| Paclitaxel | 1 (5.9) |
| Paclitaxel with bevacizumab | 1 (5.9) |
| Paclitaxel + BAY1217389 | 1 (5.9) |
| Pembrolizumab | 1 (5.9) |
| Pertuzumab with trastuzumab | 1 (5.9) |
| RX-5902 | 1 (5.9) |
| Sapanisertib with alisertib | 1 (5.9) |

S1d: Post-progression therapies for patients with metastatic TNBC.

| **TNBC** | Patients N=14* |
| --- | --- |
| Eribulin | 4 (28.6) |
| Paclitaxel | 3 (21.4) |
| RX-5902 | 3 (21.4) |
| Navelbine | 2 (14.3) |
| Carboplatin | 2 (14.3) |
| Trastuzumab | 1 (7.1) |
| Pertuzumab | 1 (7.1) |
| Ado-trastuzumab emtansine | 1 (7.1) |
| Nab-paclitaxel | 1 (7.1) |
| Atezolizumab plus ox-40 inhibitor | 1 (7.1) |
| Everolimus | 1 (7.1) |
| Capecitabine | 1 (7.1) |
| Pembrolizumab | 1 (7.1) |
| Cyclophosphamide, methotrexate, fluorouracil | 1 (7.1) |
| Doxorubicin | 1 (7.1) |
| Carboplatin with gemcitabine | 1 (7.1) |
| Sacituzumab govitecan | 1 (7.1) |
| Alisertib plus sapanisertib | 1 (7.1) |
| Ixabepilone plus capecitabine | 1 (7.1) |
| MK-1966 and SD-101 | 1 (7.1) |
| Seviteronel | 1 (7.1) |

*Number reflects patients with data on post-progression therapies and excludes patients lost to follow-up or who died prior to initiation of post-progression therapy.

S1e: All prior targeted and chemotherapy agents in HER2+ breast cancer in a curative and metastatic setting.

| **HER2+** | Patients N=2 |
| --- | --- |
| Ado-trastuzumab emtansine | 2 (100.0) |
| Capecitabine with lapatinib | 1 (50.0) |
| Entinostat with lapatinib | 1 (50.0) |
| Trastuzumab with carboplatin | 1 (50.0) |
| Trastuzumab with eribulin | 1 (50.0) |
| Trastuzumab with gemcitabine | 1 (50.0) |
| Trastuzumab with lapatinib | 1 (50.0) |
| Trastuzumab with paclitaxel | 1 (50.0) |
| Trastuzumab with vinorelbine | 1 (50.0) |
| Trastuzumab, docetaxel, carboplatin | 1 (50.0) |
| Trastuzumab, docetaxel, pertuzumab | 1 (50.0) |
| Trastuzumab, lapatinib, vinorelbine, denosumab | 1 (50.0) |
| Trastuzumab, doxorubicin, cyclophosphamide, paclitaxel | 1 (50.0) |
